# Are there too many screw holes in plates for fracture fixation?

**DOI:** 10.1186/s12893-017-0244-8

**Published:** 2017-04-21

**Authors:** Hongzhi Lv, Wenli Chang, Peizhi Yuwen, Na Yang, Xiaoli Yan, Yingze Zhang

**Affiliations:** 1 Editorial department, The Third Hospital of Hebei Medical University, NO.139 Ziqiang Road, Shijiazhuang, 050051 People’s Republic of China; 2 Department of Orthopedic Surgery, The Third Hospital of Hebei Medical University, NO.139 Ziqiang Road, Shijiazhuang, 050051 People’s Republic of China

**Keywords:** Traumatic fracture, Plate osteosynthesis, Risk factors, Implant breakage, Internal fixation

## Abstract

**Background:**

Implant breakage after the fixation of traumatic fractures is rare; however, when it occurs, it is debilitating for the patients and a challenge for surgeons. The purpose of this study was to analyze and identify the independent risk factors for implant breakage of traumatic fractures treated with plate osteosynthesis.

**Methods:**

We reviewed the medical records of patients with a fracture to any part of their four extremities, clavicle, hand or foot, who underwent surgical plate osteosynthesis from January 2005 to January 2015, and who sustained a subsequent implant breakage. Kaplan–Meier univariate and multivariate Cox regressions were performed to identify independent associations of potential risk factors for implant breakage in this cohort.

**Results:**

We identified 168 patients who underwent plate osteosynthesis surgery and had subsequent internal fixator breakage. The mean patient age was 40.63 ± 16.71 years (range, 3 to 78 years), with 72.0% (121) males and 28.0% (47) females. The average time between surgery and implant breakage was 12.85 ± 12.42 months (range, 1 to 60 months). In the final regression model, we show that inserting screws close to the fracture line is an independent predictive risk factor for implant breakage (HR, 2.165, 95%CI, 1.227 to 3.822; *P* = 0.008).

**Conclusions:**

We found that inserting screws close to the fracture line is related to an increased risk of internal fixator breakage in patients treated with plate osteosynthesis after fracture. Plates with additional holes likely lead to an increased risk of implant breakage, presumably because surgeons cannot resist inserting extra screws into the holes adjacent to the fracture line, which reduces the stiffness of the plate. We have addressed this problem by designing a plate without holes adjacent to the fracture line.

## Background

Internal fixator breakage occurs in approximately 3.5% to 13.3% of patients during internal fixation surgery follow-up [1, 2]. These complications are a challenge for even the most experienced surgeons, and can cause the patient substantial functional impairments, such as persistent and prolonged physical and psychological disabilities.

Previous studies show that risk factors for implant breakage include age, American Society of Anesthesiologists (ASA) score, fall from a height, body mass index, systemic patient comorbidities, patient postoperative noncompliance, local pathology at the fracture site, surgeons who treat a high number of patients, and surgeon technical error; this includes the use of specially designed plates rather than plates routinely used by other surgeons for treatment [3–6]. Several fixation characteristics linked with construct strength and resilience have been biomechanically evaluated and found to be related to the risk of failure, including the number of screws, the density of screws (number of screws/the number of plate holes), and the working length (plate length spanning the fracture site between two screws on each side adjacent to the fracture) [7–10]. However, there is limited data available in the literature to validate these concepts from a clinical perspective.

This study aims to investigate a large population of patients who experienced implant breakage after plate of a traumatic fracture(s) to determine the risk factors associated with this specific complication.

## Methods

### Data sources

We retrieved and reviewed the medical records of patients who were admitted to the Third Hospital of Hebei Medical University in China from January 2005 to January 2015. We included in the study patients who had any fracture to the four extremities, clavicle, hand or foot, who underwent treatment via closed reduction or open reduction and plate osteosynthesis, and who subsequently developed an implant breakage. We excluded patients who sustained direct trauma from the internally fixed fracture, those who experienced a pathological fracture, those with a psychiatric disorder, and those who suffered traumatic brain injury.

We recorded specific characteristics related to the: (1) patient (age, sex, residence, body mass index (BMI), osteoporosis, ASA classification, and medical comorbidities); (2) fracture (side of fractured limb, mechanism of injury, fractured site, fractured bones, fracture pattern, AO/OTA classification, seasonality, open/closed fracture, number of fractures); (3) surgery (shape and type of the used plate, number of used plates, ancillary fixation, number of ancillary K-wires, number of ancillary screws, inserting screws closely adjacent to the fracture line, number of empty screw holes adjacent to the fracture line, number of plate holes, number of plate screws, name and level of the surgeon performing the operation, open or closed reduction of the fracture, postoperative complication), and (4) implant breakage (breakage site within/outside the fracture line, screw slack off the hole, type of broken plate, and most possible underlying cause for the breakage).

**Fig. 1:**
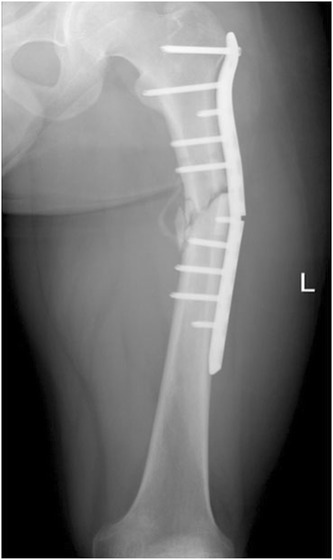
The anteroposterior radiograph of proximal femur shows the plate breakage and nonunited proximal femoral shaft fracture

All radiographs were reviewed by professors working in the Department of Orthopedic Surgery. If there were any disagreements in assessing the data, a final decision was made by discussion and consensus (Fig. 1).

### Statistical analysis

Statistical analyses were performed with SPSS21.0 (IBM, USA) software. A *P* value <0.05 was considered significant. Cox proportional-hazard regression models were used to perform survival analyses, to identify the risk factors for plate breakage, and to calculate hazard ratios (HR_S_). A univariate analysis was performed using Kaplan–Meier curves. Kaplan–Meier curves also provided a graphical comparison of survivorship for the procedures that were used over the period of the study, with the time of implant breakage as the end-point.

## Results

**Fig. 2:**
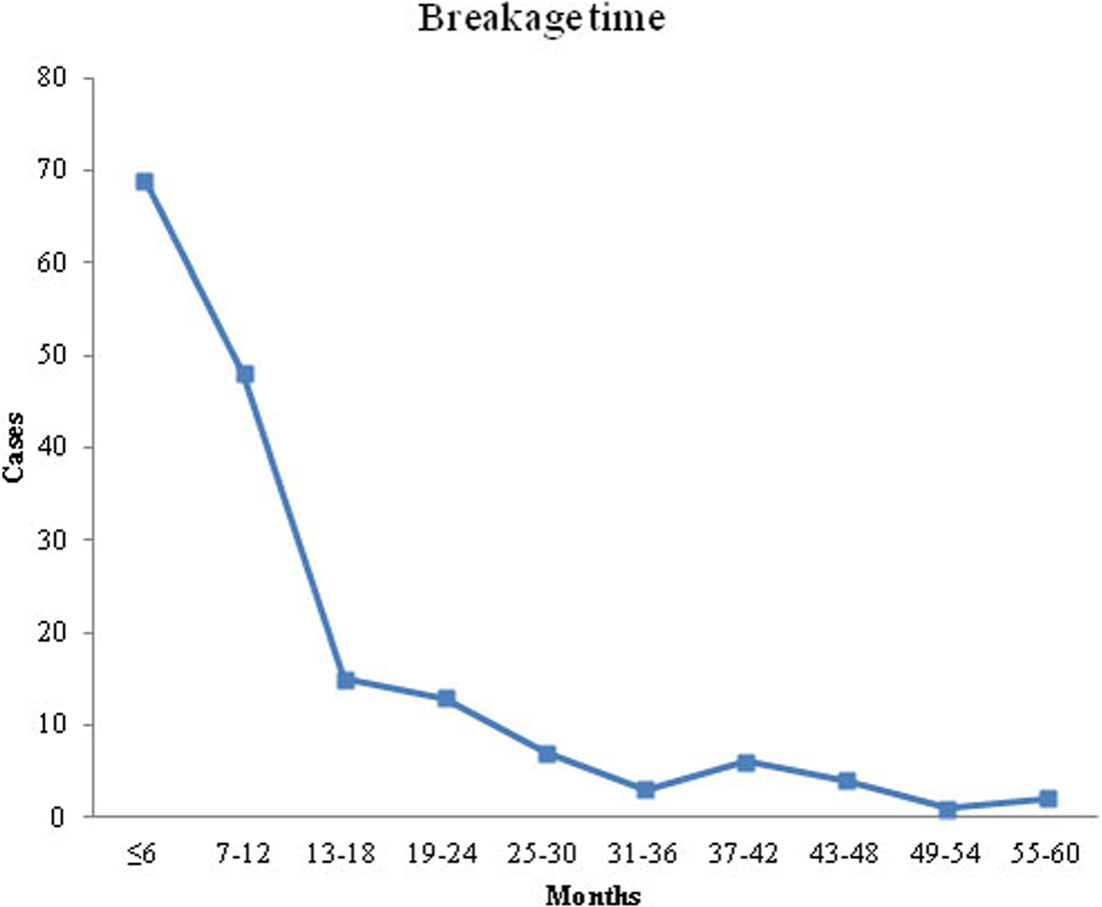
The interval between plate osteosynthesis surgery and implant breakage and the corresponding number of cases with implant breakage during the study period

Of the 201 patients identified who underwent surgery from January 2005 to January 2015, 168 patients met the inclusion criteria for the study. The mean age of the population was 40.63 ± 16.71 years (range, 3 to 78 years). Among these patients, 72% (121) were male and 28% (47) were female. The average time between internal fixation surgery and implant breakage was 12.85 ± 12.42 months (range, 1 to 60 months). Implant breakage occurred in 117 patients within less than 1 year, and this accounts for 70% of all cases (Fig. 2).

**Fig. 3:**
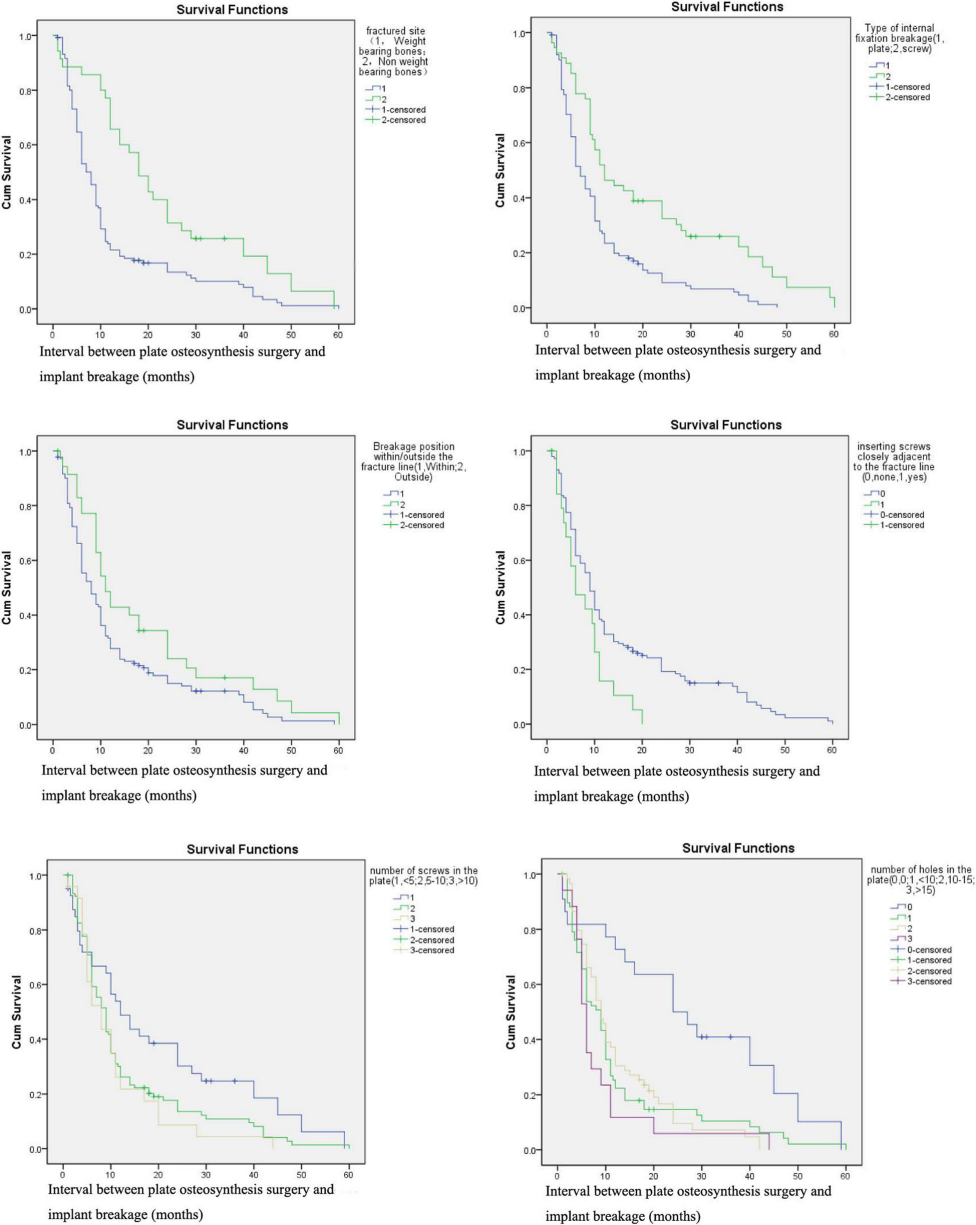
Kaplan-Meier survival curves of each of the covariates

The risk factors that were investigated are listed in Table 1. According to the Kaplan–Meier univariate analysis, implant breakage was associated with the weight or not weight limbs (*χ* ^*2*^ = 14.466, *P* < 0.001), the type of broken plate (*χ* ^*2*^ = 17.456, *P* < 0.001), breakage site within/outside the fracture line (*χ* ^*2*^ = 5.196, *P cps* 0.023), the number of plate holes (*χ* ^*2*^ = 17.119, *P* < 0.001), the number of plate screws (*χ* ^*2*^ = 6.604, *P* = 0.037), and inserting screws closely adjacent to the fracture line (*χ* ^*2*^ = 5.674, *P* = 0.017, Fig. 3).

**Table 1:** Characteristics of Patients with Internal fixation breakage

Issues	Interval between internal fixation and implant breakage, months (M (QR))	*n*(%)	*χ* ^*2*^ value	*P* value
Patients characteristics				
Age (years)			5.212	0.634
0–10	6.0	3(1.8)		
11–20	6.0(9.0)	9(5.4)		
21–30	8.0(11.2)	32(19.0)		
31–40	9.0(21.3)	44(26.2)		
41–50	10.0(8.0)	29(17.3)		
51–60	10.5(16.2)	22(13.1)		
60–69	9.0(14.0)	23(13.7)		
70+	8.5(21.2)	6(3.6)		
Gender			0.008	0.927
Male	9.0(12.5)	121(72.0)		
Female	9.0(14.0)	47(28.0)		
Body Mass Index (BMI kg/cm^2^)			5.398	0.249
< 18.5	10.0	1(0.6)		
18.5–23.9	6.5(11.0)	56(33.3)		
24.0–27.9	9.0(8.5)	56(33.3)		
28–30	10.0(17.0)	39(23.2)		
≥ 30	10.8(8.3)	16(9.5)		
Residence			2.043	0.153
Urban	7.0(9.0)	36(21.4)		
Rural	9.0(12.8)	132(78.6)		
Osteoporosis			0.746	0.388
None	9.0(12.0)	149(88.7)		
Yes	9.0(16.0)	19(11.3)		
ASA classification			2.444	0.486
1	8.0(8.0)	17(10.1)		
2	9.0(13.5)	133(79.2)		
3	8.0(17.0)	15(8.9)		
4	10.0(4.0)	3(1.8)		
Medical comorbidities			6.829	0.145
None	9.0(13.0)	141(83.9)		
Diabetes	6.0(6.0)	9(5.4)		
Hypertension	11.0(11.0)	10(6.0)		
Cardiovascular system disease	4.0(7.0)	7(4.2)		
Others*	5.0	1(0.6)		
Fracture characteristics				
Fracture pattern			3.012	0.390
Comminuted fracture(s)	9.3(14.0)	70(41.7)		
Wedge fracture(s)	6.0(8.5)	78(46.4)		
Oblique fracture(s)	14.0(23.0)	13(7.7)		
Transverse fracture(s)	10.0(26.0)	7(4.2)		
AO/OTA classification			0.103	0.950
Type A	9.0(13.5)	46(27.4)		
Type B	9.0(10.0)	88(52.4)		
Type C	8.5(14.3)	34(20.2)		
Fractured bones			3.288	0.511
Humerus	9.5(18.7)	16(9.5)		
Radius/ulna	10.0(8.0)	15(8.9)		
Femur	9.0(14.0)	87(51.8)		
Tibia/fibula	9.0(11.0)	37(22.0)		
Others (Hand, foot and clavicle)	6.0(10.0)	13(7.7)		
Weight/not weight limbs			14.466	<0.001*
Lower limb (Weight bearing bones)	7.0(7.0)	133(79.2)		
Upper limb (Non-weighted bearing bones)	18.0(18.0)	35(20.8)		
The side of fracture limbs			0.182	0.670
Left	9.0(13.0)	92(54.8)		
Right	9.5(13.0)	76(45.2)		
Seasonality			1.490	0.685
Spring	10.5(19.0)	46(27.4)		
Summer	9.5(9.0)	40(23.8)		
Autumn	7.0(9.7)	37(22.0)		
Winter	9.0(14.0)	45(26.8)		
Open/closed fracture			0.016	0.899
Open fracture	8.5(13.2)	26(15.5)		
Closed fracture	9.0(13.3)	142(84.5)		
Mechanism of injury			3.600	0.463
Motor vehicle accident	9.0(13.0)	99(58.9)		
Pedestrian fall	8.5(10.9)	44(26.2)		
Crush	11.5(21.8)	8(4.8)		
Fall from height	11.0(10.1)	14(8.3)		
Sport accident	6.0(7.0)	3(1.8)		
Number of fracture(s)			2.031	0.362
1	9.0(12.5)	145(86.3)		
2	6.0(14.5)	14(8.3)		
> 2	6.0(10.5)	9(5.4)		
Operation related issues				
Number of used plates			1.560	0.212
1	12.6(13.0)	159(94.6)		
2	17.4(25.0)	9(5.4)		
The shape of used plates			0.961	0.916
Straight	9.0(13.3)	150(89.3)		
L-shape	9.0(7.7)	8(4.8)		
T-shape	6.0(17.0)	7(4.2)		
Y-shape	24.0	1(0.6)		
O-shape	11.0	2(1.2)		
Ancillary fixation			0.427	0.935
None	9.0(10.0)	106(63.1)		
K-wire(s)	11.0(14.0)	19(11.3)		
Screw(s)	9.0(14.0)	42(25.0)		
K-wire(s) and Screw(s)	8.0	1(0.6)		
Number of ancillary K-wire(s)			0.071	0.965
0	9.0(13.0)	149(88.7)		
1	8.5(11.5)	8(4.8)		
> 1	11.0(14.0)	11(6.5)		
Number of ancillary screw(s)			7.751	0.054
0	9.0(10.0)	126(75.0)		
1	8.0(6.8)	20(11.9)		
2	9.0(24.0)	11(6.5)		
> 2	19.0(21.0)	11(6.5)		
Inserting screws closely adjacent to the fracture line			5.674	0.017*
None	9.0(13.0)	146(86.9)		
Yes	5.5(8.3)	22(13.1)		
Number of empty screw holes adjacent to the fracture line			6.245	0.182
0	5.5(8.3)	22(13.1)		
1	10.0(13.0)	76(45.2)		
2	8.5(16.3)	40(23.8)		
3	9.0(8.8)	22(13.1)		
> 4	9.0(19.5)	8(4.8)		
The type of used plate			0.122	0.727
Plate(s)	9.5(10.5)	71(42.3)		
Locked plate(s)	9.0(13.0)	97(57.7)		
Surgeon performing the operation			0.003	0.953
Chief physician	9.0(12.5)	129(76.8)		
Associate chief physician	9.0(14.0)	39(23.2)		
Open or closed reduction of the fracture			0.460	0.498
Open reduction and internal fixation	9.0(12.8)	164(97.6)		
Closed reduction and internal fixation	21.0(17.3)	4(2.4)		
Postoperative complication(s)			6.170	0.187
None	8.0(16.0)	35(20.8)		
Fracture nonunion	9.0(11.8)	112(66.7)		
Fracture delayed union	6.0(35.0)	6(3.6)		
Infection	9.0(14.5)	9(5.4)		
Others	30.0(42.8)	6(3.6)		
Postsurgical infection(s)			0.313	0.855
None	9.0(12.5)	154(91.7)		
Superficial infection	9.0(18.3)	8(4.8)		
Deep infection	7.5(18.2)	6(3.6)		
Number of plate holes			17.119	<0.001*
< 5	25.5(24.5)	22(13.1)		
5–10	8.0(8.3)	69(41.1)		
11–15	9.0(12.0)	60(35.7)		
> 15	6.0(5.5)	17(10.1)		
Number of inserted screws			6.604	0.037*
< 5	12.0(25.0)	40(23.8)		
5–10	9.0(9.0)	104(61.9)		
10	7.0(7.5)	24(14.3)		
Breakage characteristics				
Breakage site within/outside the fracture line			5.196	0.023*
Within	8.0(10.0)	132(78.6)		
Outside	11.0(17.3)	36(21.4)		
Screw slack off the hole			0.068	0.794
None	9.0(13.0)	125(74.4)		
Yes	9.0(13.0)	43(25.6)		
Type of broken plate			17.456	<0.001*
Plate(s)	6.5(8.1)	114(67.9)		
Screw(s)	12.0(19.5)	54(32.1)		
Most possible underlying cause of breakage			6.384	0.172
Internal fixator improper selection	10.0(21.8)	36(21.4)		
Premature postoperative training	10.0(15.0)	39(23.2)		
Too short of a plate utilized	7.0(8.6)	34(20.2)		
Screw(s) in inappropriate location	5.0(11.0)	39(23.2)		
Others	8.0(8.3)	20(11.9)		

*Significant at α = 0.05

**Fig. 4:**
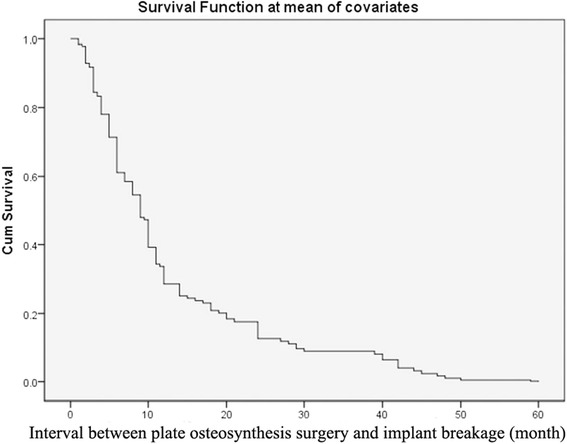
Cox survival function curves for each of the covariates

Table 2 shows the results from the multivariate Cox regression analysis, which was used to assess the association of risk factors after adjusting for all other potential risk factors. In the final model, “inserting screws adjacent to the fracture line” was identified to be independently predictive of having implant breakage (HR, 2.165; 95% CI, 1.227 to 3.822; *P* = 0.008), with a 2.165-times increased risk of causing internal fixator breakage (Fig. 4). In the study, 13.1% of surgeons did not adhere to the principle for screw placement, and inserted screws closely adjacent to the fracture line.

**Table 2:** Potential Predictors of Internal fixation breakage

Predictor	*B*	*SE*	*Wald*	*P* value	*HR*	95% *CI*		
Weight/not weight limbs	-0.533	0.315	2.859	0.091	0.587	0.316	to	1.089
Type of broken plate	-0.352	0.290	1.474	0.225	0.703	0.399	to	1.241
Breakage site within/outside the fracture line	-0.143	0.278	0.266	0.606	0.866	0.502	to	1.494
Inserting screws closely adjacent to the fracture line	0.772	0.290	7.100	0.008*	2.165	1.227	to	3.822
Number of plate hole(s)			2.855	0.414				
< 5	-0.993	0.649	2.343	0.126	0.370	0.104	to	1.321
5–10	-0.459	0.379	1.468	0.226	0.632	0.301	to	1.328
11–15	-0.442	0.331	1.781	0.182	0.643	0.336	to	1.230
Number of plate screw(s)			2.633	0.268				
< 5	0.651	0.447	2.128	0.145	1.918	0.799	to	4.604
5–10	0.194	0.317	0.374	0.541	1.214	0.652	to	2.260

*P < 0.05 through multivariate Cox regression analysis

## Discussion

Plate osteosynthesis of a fracture is a common procedure with well-established efficacy for achieving union, reducing pain, and improving function in appropriately selected patients [11–13]. However, the occurrence of plate-screw construct breakage is hard to avoid. The rate of nonunion for mid-shaft clavicle fractures is 1.4% [14], whereas that of femoral fractures is 6% to 17% [15–18]. In recent years, there has been an increase in the frequency of implant breakage along with the increasing use of plate fixation; yet, there are few studies describing the factors that contribute to this complication. The present study used a long-term follow-up of a large patient population to identify the independent risk factors associated with plate fixation breakage among patients with traumatic fractures.

Our single-factor analysis showed that the risk of fracture was associated with the weight or not weight limbs, the type of broken plate, breakage site within/outside the fracture line, the number of plate holes, the number of plate screws, and inserting screws closely adjacent to the fracture line. A multivariate Cox regression analysis confirmed that inserting screws adjacent to the fracture line was related to an increased risk of implant breakage for patients who had a fracture of the limbs, clavicle, hands, or feet. Previous studies have reported various risk factors for implant breakage, including being female, higher comorbidity scores, surgeons with fewer years’ experience, the use of longer plates, among other factors [19, 20]; we did not identify any of these factors as risk factors in our study.

In the current study, most implant breakages occurred within the first year, just as showed in Table 1. Most patients were aged between 20 and 50 years (62.5%), were male (72.0%), overweight (66.0%), and from a rural area (78.6%). Patients at risk were more likely to have experienced a high-energy trauma (73.8%) or complex fracture (88.1%) to a lower limb (79.2%), with failure occurring as a result of plate breakage (67.9%).

Plate osteosynthesis can provide relative stability, keep the fracture in a better biological position, and promote callus formation and fracture healing [21]. For complex fractures, recommendations are to use longer plates but without placing screws into the holes adjacent to the fracture line. In addition, increasing the bridging plate-work length to help distribute the stress over a larger area of the plate and thereby minimize the risk of breakage is advised. Similar rules exist for the treatment of simple and comminuted fractures, with surgeons advised against placing screws into the holes adjacent to the fracture line [22, 23]. For comminuted fractures, leaving these holes empty allows for slight movement among the fracture fragments, which is beneficial for callus formation within a reasonable scope of strain [24].

Many previous studies have shown that following biological and bridge plate techniques can obtain good radiological and functional results [25–28]. In the current study, most of the surgeons (86.9%) did not place the screws close to the fracture line; however, 13.1% of surgeons did not adhere to the principle for screw placement, and this caused an increase in the rate of plate breakage.

**Fig. 5:**
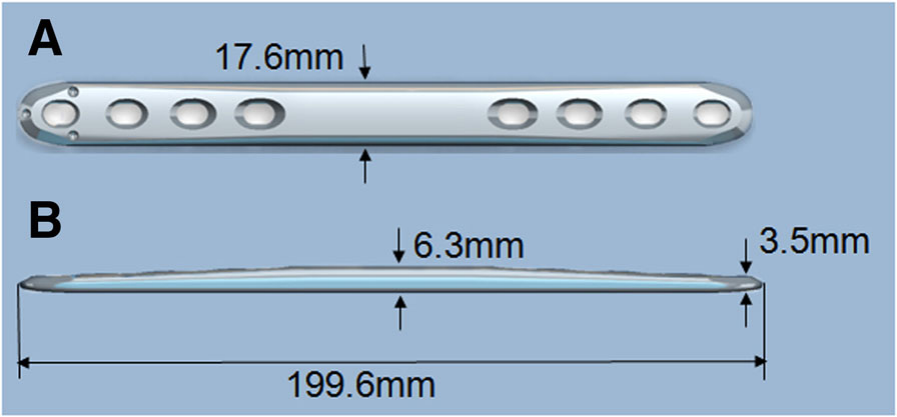
The newly-designed individual plate is featured without holes in one part of the plate, which part should be placed adjacent to the fracture line. Another is that the part without holes is the thickest, becoming thinner gradually to both ends. (**a**, anteroposterior view; **b**, lateral view)

**Fig. 6:**
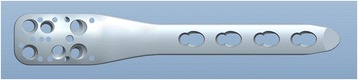
The newly-designed plate used in the fixation of surgical neck fractrue of the humerus with proximal part without holes

The presence of holes positioned adjacent to the fracture line provides an opportunity for their use, which is against recommendations. Thus, we suggest that it is unnecessary for these plates to be manufactured with these additional holes. We therefore designed a plate without holes at a part of the plate (patent number: ZL201520890025.3), and we suggest this part can be placed adjacent to the fracture line (Fig. 5). For example, surgeons can position the portion without holes in the middle for a ulnar shaft fracture, at the distal part as used in the fixation of supercondylar femoral fracture, or at the proximal part as used in the fixation of surgical neck fractrue of the humerus (Fig. 6). Another feature is that the part of the plate without holes is thickest, becoming thinner gradually to both ends (Fig. 5). This kind of plate will improve the mechanical strength of the whole plate-screw construct and subsequently reduce the risk of implant breakage.

### Limitations

Our study has a few limitations. First, this was a retrospective study, and inevitable recall bias exists. Second, we did not distinguish between bridge plating fixation and compression fixation because both principles were used in many of the cases and they are difficult (and somewhat unnecessary) to distinguish. Third, it is not certain precisely when the construct breakage occurred, with breakage time determined as the time of the latest radiographic evidence.

## Conclusions

Using multivariate Cox regression analysis, we show that there is an increased risk of implant breakage in patients who had a fracture of the four extremities, clavicle, hand or foot after plate osteosynthesis fixation when screws were placed in the holes of the plate adjacent to the fracture line. The data also suggests that the newly-designed individual plates without holes in a part of the plate, which part should be placed adjacent to the fracture line, can help reduce the risk of implant breakage. Additional prospective studies are warranted to compare this new plate type with existing instrumentation to confirm that the placement of screws near the fracture line affect the treatment of traumatic fractures.

## Abbreviations

ASA: American Society of Anesthesiologists
BMI: Body mass index
HR_S_: Calculate hazard ratios
